# Ectodermal Dysplasia: A Genetic Review

**DOI:** 10.5005/jp-journals-10005-1165

**Published:** 2012-12-05

**Authors:** Seema Deshmukh, S Prashanth

**Affiliations:** Senior Lecturer, Department of Pediatric and Preventive Dentistry, JSS Dental College and Hospital, Mysore, Karnataka, India e-mail: dr_seemadeshmukh07@yahoo.co.in; Senior Lecturer, Department of Pediatrics and Preventive Dentistry, JSS Dental College and Hospital, JSS University, Mysore Karnataka, India

**Keywords:** Ectodermal dysplasia, Anodontia, Hypodontia, Hypohidrosis, Hypotrichosis, EDA gene, EDAR gene

## Abstract

Ectodermal dysplasia is a rare hereditary disorder with a characteristic physiognomy. It is a genetic disorder affecting the development or function of the teeth, hair, nails and sweat glands. Depending on the particular syndrome ectodermal dysplasia can also affect the skin, the lens or retina of the eye, parts of the inner ear, the development of fingers and toes, the nerves and other parts of the body. Each syndrome usually involves a different combination of symptoms, which can range from mild to severe. The history and lessons learned from hypohidrotic ectodermal dysplasia (HED) may serve as an example for unraveling of the cause and pathogenesis of other ectodermal dysplasia syndromes by demonstrating that phenotypically identical syndromes can be caused by mutations in different genes (EDA, EDAR, EDARADD), that mutations in the same gene can lead to different phenotypes and that mutations in the genes further downstream in the same signaling pathway (NEMO) may modify the phenotype quite profoundly. The aim of this paper is to describe and discuss the etiology, genetic review, clinical manifestations and treatment options of this hereditary disorder.

**How to cite this article:** Deshmukh S, Prashanth S. Ectodermal Dysplasia: A Genetic Review. Int J Clin Pediatr Dent 2012; 5(3):197-202.

## INTRODUCTION

The skin with its appendages is an important organ in all higher organisms. It constitutes the primary barrier delineating self from environmental challenges. The development of such versatile and complicated structure requires a highly coordinated interaction of several genetic signaling pathways within and between the ectodermal and underlying (ecto) mesenchymal layers of embryonic tissue. Malfunction in any part of this system or at any point during development of ectodermal structures can lead to a variety of phenotypically distinctive entities which we classify as the ectodermal dysplasia syndromes.^1^

The term ectodermal dysplasia is used to designate a heterogenous group of disorders characterized by a constellation of findings involving a primary defect of the skin, teeth and appendageal structures including hair, nail, exocrine and sebaceous glands.^2^

Freire-Maia described 117 possible varieties of ectodermal dysplasia involving all possible Mendelian modes of inheritance.^3^ It was redefined by Freire-Maia as a pathogenic developmental defect, which at the embryologic level, affects the ectoderm and therefore the tissues and structures derived from it. Thus, it affects the epidermis, in which it is responsible for development of keratinocytes and causes abberations in the hair, sebaceous glands, eccrine and apocrine glands, nails, teeth and lenses conjunctiva of eye, anterior pituitary gland, nipples and the ear. In addition there are defect of central nervous system (CNS), the adrenal medulla the oral, nasal and rectal mucosa and their associated glands. The pharyngeal and laryngeal mucosa may be so, atrophic that it results in dysphonia and hoarseness of voice.^4^

## HISTORIC PREVIEW

According to Perabo et al ectodermal dysplasia may have been recorded as early as 1792 by Danz. In 1838, Wedderburn documented ectodermal dysplasia in a letter to Charles Darwin, describing a case of 10 Hindu male family members. Thurnam in 1848 reported 2 cases of hypohidrotic form. Similar cases were reported by Guilford and Hutchinson in 1883 and 1886 respectively. Weech, in 1929 introduced the term hereditary ectodermal dysplasia and suggested the term anhidrotic for those with inability to perspire. Felsher, in 1944, changed the adjective anhidrotic to hypohidrotic because the author agreed that person with hypohidrotic form are not truly devoid of sweat glands.^5,6^

## ETIOLOGY AND FREQUENCY

Hypohidrotic ectodermal dysplasia (HED) is usually transmitted as an X-linked recessive trait in which the gene is carried by the female and manifested in male.^7^ In X-linked form carrier mothers exhibit minimal expression in the form of hypodontia and/or conical teeth and spottily reduced sweating. The unaffected female has 50% chance of transmitting this disorder to her male children and each female offspring has 50% chance of inheriting the defective gene, thereby being a carrier. Spontaneous gene mutation is possible and may occur in family without any previous history of the syndrome. The prevalence in the population has been assessed as between 1:10,000 and 1:100,000 male live births.^8^

The different types of ectodermal dysplasia are caused by the mutation or deletion of certain genes located on different chromosomes. Because ectodermal dysplasias are caused by a genetic defect they may be inherited or passed down the family line. In some cases, they can occur in people without a family history of the condition, in which case a *de novo* mutation would have occurred. Mutations in the EDA, EDAR and EDARADD genes cause HED. EDA is the only gene known to be associated with X-linked HED (XLHED). Ninety-five percent of individuals with HED have the X-linked form. The genes EDAR and EDARADD are known to be associated with both autosomal dominant and autosomal recessive forms of HED. Mutations in these genes account for 5% of HED.

### Genetic Pathogenesis

The EDA, EDAR and EDARADD genes provide instructions for making proteins (ectodysplasin A) that work together during embryonic development. Ectodysplasin A forms a part of a signaling pathway that is critical for the interaction between two cell layers, the ectoderm and the mesoderm. In the early embryo, these cell layers form the basis for many of the body organs and tissues. Ectoderm-mesoderm interactions are essential for the formation of several structures that arise from the ectoderm, including the skin, hair, nails, teeth and sweat glands. Mutations in the EDA, EDAR or EDARADD gene results in defective ectodysplasin A formation thereby preventing normal interactions between the ectoderm and the mesoderm and hence impairing the normal development of hair, sweat glands and teeth. The improper formation of these ectodermal structures leads to the characteristic features of hypohidrotic ectodermal dysplasia.^9^

### Classification of Ectodermal Dysplasia

Currently there are about 150 different types of ectodermal dysplasias. In an attempt to classify these, different subgroups are created according to the presence or absence of the four primary ectodermal dysplasia (ED) defects:

- ED1: Trichodysplasia (hair dysplasia)
- ED2: Dental dysplasia
- ED3: Onychodysplasia (nail dysplasia)
- ED4: Dyshidrosis (sweat gland dysplasia)

Based on the above, the 150 different types of ectodermal dysplasias are categorized into one of the following subgroups made up from the primary ED defects:

- Subgroup 1-2-3-4
- Subgroup 1-2-3
- Subgroup 1-2-4
- Subgroup 1-2
- Subgroup 1-3
- Subgroup 1-4
- Subgroup 2-3-4
- Subgroup 2-3
- Subgroup 2-4
- Subgroup 3
- Subgroup 4

The most common ectodermal dysplasias are hypohidrotic (anhidrotic) ectodermal dysplasia which falls under subgroup 1-2-3-4 and hydrotic ectodermal dysplasia which comes under subgroup 1-2-3. The three most recognized ectodermal dysplasia syndromes fall into the subgroup 1-2-3-4, as they show features from all four of the primary ectodermal dysplasia defects. They are:

- Ectrodactyly-ectodermal dysplasia-clefting syndrome (EEC)
- Rapp-Hodgkin HED
- Ankyloblepharon, ectodermal defects, cleft lip/palate (AEC) or Hay-Wells syndrome.^9^

## CLINICAL MANIFESTATIONS

The disorder probably appears during the first trimester of pregnancy. If it is severe, it appears before the 6th week of embryonic life, and consequently the dentition will be affected. After 8th week, the other ectodermal structures will be affected.^5^

The most remarkable characteristic of hereditary ectodermal dysplasia is hypohidrosis, because other physical features are not as apparent in the first year of life. It may be diagnosed clinically before the second year of life only after repeated episodes of unexplained fever. The inability to sweat results in intolerance to heat, occasionally causing severe incapacitation and hyperpyrexia after only mild exertion or even following just a meal.^10,17^

### Extraoral Findings

Because of partial or complete absence of sweat and sebaceous glands, the phenotype has smooth, soft, dry and thin skin. Fine linear wrinkles and increased pigmentation are often present around the eyes and mouth. There may be hyperkeratosis of the palms of the hands and soles of the feet. There is absence of lanugo hair, although beard is usually normal, axillary and pubic hair is generally sparse. The hair over the scalp is often blonde, fine, stiff and short. While the eyebrows and eyelashes are frequently missing. The nails appear to be normal or somewhat spoon shaped. In females, the mammary glands are aplastic or hypoplastic. Impaired lacrimal gland function and occasionally glaucoma has been reported. Also there can increased susceptibility to allergic disorders, such as asthma or eczema.^4^

Other manifestations include variations in the shape of the skull which can resemble inverted triangle. The face looks smaller because of the frontal bossing and depression of the nasal bridge. The lips can be protuberant and the ears may be situated obliquely on the head causing them to stand out.^4^

### Dental Manifestations

The most striking feature is oligodontia of the primary and permanent teeth. However, congenital absence of the primary teeth is relatively rare.^10^ Teeth that are present have abnormal crown form. Teeth in anterior region of the maxilla and mandible are conical shaped.^5^ The number of missing teeth varies with higher incidence in mandible. In the primary dentition, maxillary 2nd molars, canines, central incisors and mandibular canines are the teeth commonly present.^12,13^ Children with ectodermal dysplasia show maxillary retrusion due to sagittally underdeveloped maxilla, forward and upward displacement of mandible and collapsed anterior facial height. The palatal arch is frequently high and cleft palate may be present.^4,8,14^

## DIAGNOSIS

Diagnosis is based on the episodes of hyperpyrexia, lack or type of the hair, absence of teeth and tooth buds and tooth morphology. Peeling of the skin at birth, eczema, asthma, and frequent respiratory infections may be additional clues. However, during early infancy diagnosis is difficult because manifestations involving teeth, hair and inability to sweat are hard to detect.^8^ However, attempts have been made in past to develop objective diagnostic criteria based on number and distribution of sweat pores and amount of sweat produced. The structural and biochemical characteristics of hair have also been studied.^15^

Other diagnostic criteria have been dermatoglyphic analysis, characteristics of lacrimal secretion and distribution and pattern of scalp hair. Missing teeth can also be an important sign.^16^

### Laboratory Investigations for Ectodermal Dysplasia

Patients with ectodermal dysplasia associated with immunodeficiency may have hypogammaglobulinemia with impaired lymphocyte proliferation and cell-mediated immunity. An appropriate evaluation, including determination of quantitative immunoglobulin levels and T-cell subset populations, can be performed. Other tests include-Sweat pore counts, pilocarpine iontophoresis and skin biopsy which may document hypohidrosis and a reduction in the number of eccrine glands. Sweat pore counts may be performed using yellow starch—iodine powder applied to palmar or dorsal skin. In unaffected persons, sweating turns the yellow starch— iodine powder to deep purple, allowing visualization of sweat pores. Sweat pores are poorly visualized in affected children. Female carriers of X-linked disorder may demonstrate a mosaic pattern of areas of normal numbers of sweat pores alternating with areas of absent pores. Streaky areas of hypohidrosis that follow Blaschko lines are observed upon starch—iodine staining.

Prenatal diagnosis using genetic mutation analysis may be performed. Indirect prenatal diagnosis may be performed by linkage analysis applied to chorionic villus samples at the 10th week of gestation for some ectodermal dysplasias. Genetic testing for HED, X-linked recessive and autosomal dominant HED, EEC syndrome and AEC syndrome is available through GeneDx.^9^

**Table 1:** **Table 1:** Summary of molecular genetic testing used in HED

*Gene symbol*		*% of HED attributed to mutations in this gene*		*Test method*		*Mutation detected*	
EDA		95		Sequence analysis		Sequence variant	
				Deletion/duplication analysis		Partial or whole EDA deletion	
EDAR		5		Sequence analysis		Sequence variant	
EDARADD		5		Sequence analysis		Sequence variant	

## MOLECULAR GENETIC TESTING

- EDA is the only gene known to be associated with XLHED. Ninety-five percent of individuals with HED have the X-linked form.
- The genes EDAR and EDARADD are known to be associated with both autosomal dominant and autosomal recessive forms of HED. Mutations in these genes account for 5% of HED.

### Clinical Testing

Sequence analysis:

- *EDA:* In males with X-linked HED, direct sequencing of the eight exons with flanking intron sequences of EDA identifies approximately 95% of mutations, including missense and nonsense mutations and smaller deletions (Table 1).
- *EDAR:* Sequence analysis of the EDAR coding region is available on a clinical basis.
- *EDARADD:* Sequence analysis of the EDARADD coding and flanking intronic regions is available on a clinical basis.
- *Duplication/deletion testing:* Sequence analysis of EDA cannot detect exonic, multiexonic or whole gene deletions in females, and additional testing using methods that detect deletions are required.^9^

### Risk to Family Members—XLHED

Parents of a Male Proband

- The father of an affected male is neither affected nor is he a carrier.
- In a family with more than one affected individual, the mother of an affected male is an obligate carrier.
- Clinical examination may detect minimal manifestations of XLHED in the mother. Molecular genetic testing is indicated.
- When an affected male represents a simplex case (male with no known family history of XLHED), several possibilities regarding his mother’s carrier status need to be considered:
  – He has a *de novo* disease-causing mutation in the EDA gene and his mother is not a carrier.
  – His mother has a *de novo* disease-causing mutation in the EDA gene, either (a) as a ‘germline mutation’ (i.e. occurring at the time of her conception and thus present in every cell of her body); or (b) as ‘germline mosaicism’ (i.e. occurring in a certain percentage of her germ cells only).
  – His maternal grandmother has a *de novo* disease- causing mutation in the EDA gene.

Parents of a Female Proband

- The father of a female proband may be affected.
- The proband may have inherited the gene mutation from her mother.
- The proband may have a *de novo* mutation in the EDA gene.
- Clinical examination may clarify the status of the parents.

Siblings of a Proband

- The risk to siblings depends on the genetic status of the parents.
- If the mother is a carrier, the chance of transmitting the EDA mutation in each pregnancy is 50%. Male siblings who inherit the mutation will be affected; female siblings who inherit the mutation will be carriers and may show minimal manifestations.
- If the mother is not a carrier, the risk to siblings is low but greater than in the general population because the risk of germline mosaicism in mothers is not known.
- If the father is affected, none of the male siblings and all of the female siblings will inherit the mutation. The females may show minimal manifestations.

## GENETIC TRANSMISSION

### Offspring of a Male Proband

- A male with XLHED will transmit the disease-causing EDA allele to all of his daughters and none of his sons.
- The daughters will be obligate carriers and may show minimal manifestations.

### Offspring of a Female Proband

- A female with XLHED will transmit the disease-causing EDA allele to half of her children, regardless of gender. Thus, her sons have a 50% risk of being affected and her daughters have a 50% risk of being carriers, who may show minimal manifestations.

## THERAPEUTIC ASPECTS

Patients afflicted with a hereditary deformity, such as ectodermal dysplasia suffer from poor psychological and physiologic development as a result of unacceptable esthetics and abnormal functions of orofacial structures.^6^ Hence, early treatment is suggested. Optimal treatment requires a multidisciplinary collaborative efforts pediatric professionals, psychologist, ENT specialist and speech therapist. Dentists have responsibility to rehabilitate these patients to improve appearance, mastication and speech. Dental treatment depends on severity of the disorder. Therefore, treatment varies according to age, growth and development of stomatognathic system of the patient. Nowak suggests that the pediatric dentist is best suited to manage the pediatric patient’s special needs.^18^ The major goal of pediatric dentist is to provide the child with an oral apparatus that provides optimal esthetics and function to allow the patient to develop physically, emotionally and socially.^10^

Dental treatment is ongoing and active process that must be adapted constantly to the child’s growth and development. The usual pattern of treatment consists of fabrication of dentures, and as the child grows the denture will be modified or replaced. During the mixed dentition stage, the prosthesis will require modification to accommodate loss of exfoliated primary teeth and appearance of newly erupted permanent teeth. During the permanent dentition, the removable prosthesis may be replaced by fixed depending on the number and position of the teeth. Before providing the patient with the dentures, the tone of the perioral muscles can be trained by vestibular shield. Studies have shown that early prosthetic treatment helps in establishing normal craniofacial growth. It also improves harmonization of the position of the lips in relation to nose and chin.^11,13,18^

The other treatment option is the use of implants. Use of implants in adult patients is well-documented in literature, but there are limited reports of the use of implants in growing children. There are two primary concerns about the use of implants in children. First, if implants are present during several years of facial growth, do they have a danger of becoming embedded, relocated or displaced as the jaw grows? Any of these outcomes is possible because implants in contrast to teeth, are not capable of compensatory eruption or other physiologic movement. The second area of concern is the effect of prosthesis on growth.^19^ Although use of implants is most desirable, but it may not be favorable in growing children, because, a fixture in an acceptable position at 7 or 8 may not in favorable position at 16. Hence, it is recommended to consider postponing placement in children younger than 13. However, when placement of implants is the treatment of choice, bone grafting and sinus membrane elevation can be considered to facilitate placement.^19,20^ However, dental implants have proven successful in children overage 7 years only in the anterior portion of the mandibular arch.^22^

## FUTURE TREATMENT PERSPECTIVE USING GENE THERAPY

Recombinant EDA–A1 administration provides the basis for possible treatment in XLHED. Recombinant proteins containing the receptor binding domain of EDA fused to the C–terminus of IgG 1 Fc domain have been engineered to cross the placental barrier and administered to pregnant mice. This treatment permanently rescued the phenotype in the offspring. The jaw and the molars regained the normal sizes. In experimental studies both reversion of oligodontia and dental dysmorphologies were obtained by postnatal single or multiple EDA–A1 intravenous administration in XLHED dogs. The present model developed to approach the conditions of the future therapeutic use of recombinant EDA– A1 in humans, could show the ability of recombinant EDA– A1 to correct the pathological features of XLHED.^23^

## DISCUSSION

Children with congenital or craniofacial defect are unique, and oral problems must be evaluated individually to provide the most ideal treatment. Early prosthetic treatment in children with ectodermal dysplasia is important. This results in significant improvement in esthetics, masticatory and phonetic function. In addition, the positive psychological impact on the child and his/her parents should be taken into account. It has been reported that the child’s self image is fairly completed by 4 to 5 years.^5^ Research has shown that global self-esteem in children and adolescents is highly determined by assessment of one’s own physical presentation, as well as comparison with the attractiveness, ability, intellectual skills and social acceptance of other people. Unusual facial features exacerbate the social challenges of meeting new people. Lowered self-esteem, speech defects, decreased academic performance and social isolation may result from merely looking different from one’s peers.^21^

Therefore, cosmetic and prosthodontic measures should be instituted as early as possible to have the child resemble his peers. It is also important to instill awareness among parents regarding early management. To ensure adequate care, child with ectodermal dysplasia should be managed by a team which includes pediatrician, pediatric dentist, prosthodontist, dermatologist, otolaryngologist, speech therapist and psychologist. However, an appropriate change has to made in the denture so that the appearance is always appropriate for the age. Relining or change of dentures should be done every 1 or 2 years due to preadolescent growth in jaw dimensions, wear of the acrylic teeth, under extension of the dentures and posterior open bite. Cephalometric analysis has shown favorable growth of maxilla and mandible following the placement of dentures.^5^

Recently, implant borne total telescopic dentures have been described as a possible treatment strategy. But high- cost difficulties in placement and high failure rate make their use questionable. Analogous to the ankylosed tooth implants cannot erupt and are likely to impede or even stop the normal growth. Therefore, option is not recommended in children before skeletal maturity.^18^

However, the successful use of any prosthesis depends on the cooperation and communication between members of dental team and the patient.
